# Associations Among Menstrual Cycle Length, Coronavirus Disease 2019 (COVID-19), and Vaccination

**DOI:** 10.1097/AOG.0000000000005343

**Published:** 2023-08-10

**Authors:** Alexandra Alvergne, Emily Boniface, Blair Darney, Amanda Shea, Kirsten Weber, Cécile Ventola, Virginia J. Vitzthum, Alison Edelman

**Affiliations:** Institute for Evolutionary Sciences, Montpellier University, Montpellier, France; the School of Anthropology and Museum Ethnography, University of Oxford, Oxford, United Kingdom; the Department of Obstetrics and Gynecology, Oregon Health & Science University, and the OHSU-PSU School of Public Health, Portland, Oregon; the National Institute of Public Health (INSP), Center for Population Health (CISP), Cuernavaca, Morelos, Mexico; and Clue by BioWink GmbH, Berlin, Germany.

## Abstract

**OBJECTIVE:**

To assess whether coronavirus disease 2019 (COVID-19) is associated with menstrual cycle length changes and, if so, how that compares with those undergoing vaccination or no event (control).

**METHODS:**

We conducted a retrospective cohort analysis in which we analyzed prospectively tracked cycle-length data from users of a period tracker application who also responded to a survey regarding COVID-19 symptoms and vaccination. We restricted our sample to users aged 16–45 years, with normal cycle lengths (24–38 days) and regular tracking behavior during the five cycles around COVID-19 symptoms or vaccination or a similar time period for those experiencing no event (control group). We calculated the within-user change in cycle length (days) from the three consecutive cycles preevent average (either vaccination, disease, or neither; cycles 1–3) to the event (cycle 4) and postevent (cycle 5) cycles. We used mixed-effects models to estimate the age- and country-adjusted difference in change in cycle length across the groups.

**RESULTS:**

We included 6,514 users from 110 countries representing 32,570 cycles (COVID-19 symptoms: 1,450; COVID-19 vaccination: 4,643; control: 421). The COVID-19 cohort experienced a 1.45-day adjusted increase in cycle length during cycle 4 (COVID-19) compared with their three preevent cycles (95% CI 0.86–2.04). The vaccinated group experienced a 1.14-day adjusted increase in cycle length during cycle 4 (COVID-19 vaccine) compared with their preevent average (95% CI 0.60–1.69). The control group (neither vaccine nor disease) experienced a 0.68-day decrease (95% CI −1.18 to −0.19) in a similar time period. Post hoc tests showed no significant differences in the magnitude of changes between the COVID-19 and vaccination cohorts. In both cohorts, cycle length changes disappeared in the postevent cycle.

**CONCLUSION:**

Experiencing COVID-19 is associated with a small change in cycle length similar to COVID-19 vaccination. These changes resolve quickly within the next cycle.

## Abstract 2

Experiencing coronavirus disease (COVID-19) is associated with a small change in menstrual cycle length that is similar to COVID-19 vaccination.

The coronavirus disease 2019 (COVID-19) pandemic has revealed many significant knowledge gaps, notably a lack of information regarding the potential effects of vaccines and infection on the menstrual cycle.^1–3^ Menstruation is increasingly being recognized as a critically important patient-reported outcome,^4–6^ in part due to the initial scrutiny placed on the association of COVID-19 vaccines and menstrual cycle disturbances.^7–15^ Yet, as the prevalence of COVID-19 has increased, growing reports of menstrual disturbances after severe acute respiratory syndrome coronavirus 2 (SARS-CoV-2) infection^16,17^ and with long-COVID^18^ are occurring.

The immune and reproductive systems are known to interact with each other,^19,20^ and temporary disruption of the menstrual cycle is seen with acute infection and febrile episodes. However, previous literature on the association between COVID-19 and menstrual cycle changes is scarce and inconsistent, limited by small samples, recall bias, lack of comparison (unexposed) groups, or small subgroups of participants with COVID-19.^21–23^

Menstrual cycles have their own normal inherent variability,^24^ which makes it particularly challenging to determine whether an exposure causes a change without access to prospectively collected population-level data before and after the exposure. Similar to prior work,^8–10^ we present an analysis of prospectively collected menstrual cycle tracking data to assess whether COVID-19 is associated with changes in cycle length. We compared within-individual changes in cycle length among groups: 1) a COVID-19 group, 2) a COVID-19–vaccinated group, and 3) a control group of unvaccinated participants reporting no history of COVID-19.

## METHODS

We conducted a retrospective cohort analysis of menstrual cycle data collected prospectively by users of the period tracker application (app) Clue, linked to survey data on COVID-19 vaccination and disease status. The study received clearance from the French Research Institute for Development Ethic Board (IRD CCERP), Montpellier University Ethic Board, and the Oregon Health & Science University IRBs.

Only users aged 16–58 years with a registered period tracker app account and who gave consent for their pseudonymized data to be used for research purposes were sent an in-app message to take part in the survey, “Period and the Pandemic.” The survey distributed to period tracker app users in the United States, the United Kingdom, Canada, and Australia between November 29, 2021, and February 8, 2022, and then to all period tracker app users with their app set to English-language between May 19, 2022, and August 10, 2022. We stopped data collection when no new entries were recorded. The link to the survey was seen by 3,310,221 users, and 443,134 clicked on the link, leading to a clickthrough rate of 13.4%, consistent with typical response rates for in-app surveys.

After giving consent to link their survey and their prospectively collected menstrual cycle data (starting in 2019), users completed the survey questions regarding their COVID-19 vaccination status and dates of vaccination and COVID-19 history (month and year of onset of first symptoms or date of a positive test result), as well as age, body mass index (BMI, calculated as weight in kilograms divided by height in meters squared), and country of location. To evaluate the independent associations of disease and vaccination with cycle length, we created three groups: 1) a control group, including participants with no history of either COVID-19 vaccination or having COVID-19; 2) a vaccinated group, including participants with a history of COVID-19 vaccination but no history of having COVID-19 and for whom the first vaccine occurred in the first 38 days of the cycle and the second vaccine occurred at least 38 days after the first vaccine (to avoid the potential effects of a second vaccine dose); and 3) a COVID-19 group, including participants with a history of having COVID-19 and reporting being either unvaccinated or vaccinated at least 76 days (ie, two cycles of 38 days) after the onset of COVID-19 symptoms.

We excluded individuals who reported hormonal contraceptive use at any time between 2019 and the time of the survey. Data on contraception were taken either from the survey when available or from data tracked by users within the app during the study period. We also excluded users older than age 45 years, those for whom no cycle data were available, those who did not report their COVID-19 and vaccination status, and those who gave inconsistent or no dates for COVID-19 symptoms or vaccination. We removed all cycles flagged by users as abnormal (n=10,788).

**Fig. 1.**
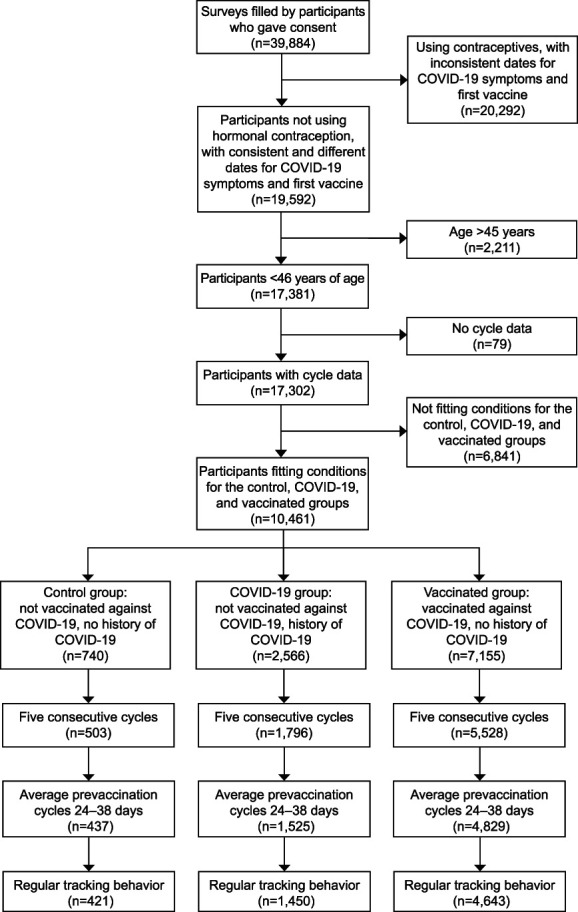
STROBE (Strengthening the Reporting of Observational Studies in Epidemiology) flow diagram. COVID-19, coronavirus disease 2019.

We included users with at least five consecutive cycles. For the control group, we selected the last three cycles of 2020 (cycles 1–3) as artificial preevent cycles and two post–first artificial event cycles, inclusive of the event cycle (cycle 4). The artificial event was the first cycle of 2021 that contained January 31, 2021, to reduce bias from asymptomatic cases and align with the timing of most vaccinations. We used alternative dates (October 31, 2019, January 31, 2020, or October 31, 2020) in sensitivity analyses. For the vaccinated group, we included three prevaccine cycles (cycles 1–3) and two post–first vaccine cycles, inclusive of the vaccination cycle (cycle 4). For the COVID-19 group, we included three pre-COVID cycles (cycles 1–3) and two post–first COVID cycles, inclusive of the COVID cycle (cycle 4). When the month but not the day of COVID-19 symptom onset was recorded, we took the cycle that overlapped the most with the month during which COVID-19 symptoms occurred. For all three groups, we excluded all participants with average preevent cycles outside the 24–38 day range for cycle length.^25^ To reduce the possibility that missing data increased the length of the event cycle, we excluded all participants who did not track at least one symptom of any type every 38 days in the 90 days after the start of cycle 4, so that an absence of bleeding in this period cannot be attributed to nonadherence with tracking (Fig. 1).

*Alvergne. Menstrual Cycle Length and COVID-19. Obstet Gynecol 2023.*

Our primary exposure was self-reported vaccination and disease status (ie, group). Our primary outcome was the within-user change in cycle length (days) from the three-cycle preevent average (cycle 1–3) to the event cycle (cycle 4). Our secondary outcome was the within-user change in cycle length (days) from the three-cycle preevent average (cycle 1–3) to the first postevent (cycle 5). Our third outcome was the proportion of users who experienced a clinically significant change in cycle length (more than 8 days) in cycles 4 and 5.^25^

Assuming an SD of 4 days and a minimum sample size of 421 per group, we had more than 90% power to detect an unadjusted 1-day within-individual difference in cycle length given three groups, lowering the significance level from 0.05 to 0.0125 to adjust for multiple outcomes (Appendix 1, available online at http://links.lww.com/AOG/D312). All analyses were conducted using R 4.2.1.^26^ We compared within-individual changes in cycle length between the three-cycle preevent average (cycles 1–3) and cycles 4 and 5 using a two-sided *t* test. We determined outliers using the Cook's distance method and excluded them for the analysis (Appendix 2, available online at http://links.lww.com/AOG/D312). We created histograms to compare the distributions of changes in cycle length and compared the proportion of individuals who experienced a clinically significant change in cycle length (8 days or more)^25^ using Pearson's χ^2^ tests. We used longitudinal multivariable mixed-effects models to determine the adjusted difference in the change in cycle length among groups and plotted predicted values. Models contained random intercepts and slopes at the individual level and interaction term between time (preevent cycle average, event cycle, and postevent cycle) and group (control, COVID-19, COVID-19 vaccination). All estimates were adjusted for age and country, and *P* values were adjusted for multiple comparisons using a Bonferroni–Holm correction to control the family-wise error rate. A post hoc Tukey honestly significant difference (HSD) test was used to assess the significance of differences between pairs of group means.

Age and country data were available for the full selected sample, but information on BMI (categorized as underweight, normal weight, overweight, or obesity) was missing for most participants (94.6%; Appendix 3, available online at http://links.lww.com/AOG/D312). To avoid reducing statistical power, our complete cases analysis did not include BMI as a covariate. We then imputed data on BMI using a multiple imputation approach,^27^ combining random forest imputation with predictive mean matching (Appendix 3, http://links.lww.com/AOG/D312), and conducted a sensitivity analysis.

We also explored whether vaccination before the onset of COVID-19 symptoms mediated any association between COVID-19 and cycle length. We created an additional group including participants reporting vaccination at least 3 months before the onset of COVID-19 symptoms and reran all steps of the analysis, including removing outliers and adjusting for age and country (Appendices 4–6, available online at http://links.lww.com/AOG/D312).

We conducted three sensitivity analyses to confirm the robustness of our findings: 1) we excluded individuals who reported COVID-19 symptoms after November 2021 to rule out any effect of the Omicron wave (Appendix 7, available online at http://links.lww.com/AOG/D312), 2) we performed a sensitivity analysis including imputed data on BMI (Appendix 8, available online at http://links.lww.com/AOG/D312), and 3) we changed the timing of the artificial COVID cycle in the control group (Appendix 9, available online at http://links.lww.com/AOG/D312).

## RESULTS

Of 39,884 eligible individuals, 6,514 met inclusion criteria (Fig. 1). The final study sample included 6,514 participants representing 32,570 cycles (five cycles per individual), including 421 individuals in the control group, 1,450 in the COVID-19 group, and 4,643 in the COVID-19 vaccinated group (Table 1). Initial COVID-19 symptoms occurred between January 1, 2020, and June 28, 2022, and initial COVID-19 vaccinations occurring between December 11, 2020, and July 26, 2022 (Appendix 10, available online at http://links.lww.com/AOG/D312). Included vaccine types were Pfizer-BioNTech, Oxford-AstraZeneca, CoronaVac/Sinovac, Covishield, Johnson & Johnson/Janssen, Moderna, Sinopharm, and Sputnik V. Participants were from 110 countries (Appendix 11, available online at http://links.lww.com/AOG/D312), with most participants coming from the United States (3,237, 49.7%), the United Kingdom (922, 14.1%), Germany (324, 5.0%), Canada (235, 3.6%), France (206, 3.2%), and Australia (206, 3.2%)

**Table 1.**
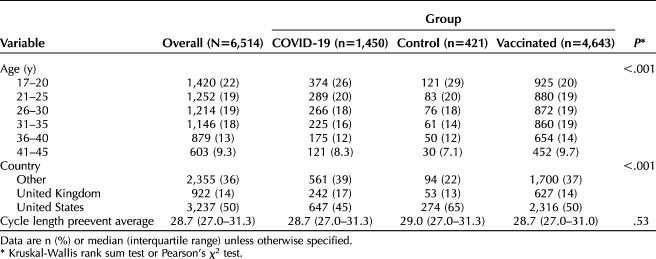
Sample Characteristics

**Fig. 2.**
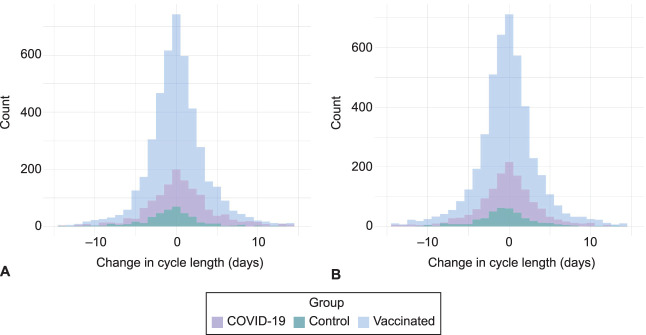
Distribution of changes in cycle length between the three preevent cycles average and the event cycle (**A**) and the postevent cycle (**B**). This includes data from people who tracked any symptom at least every 38 days for 90 days from the start of the event cycle and excludes outliers (n=6,396). The proportion of individuals with a cycle change more than 15 days is 1.6% in the event cycle (**A**) and 2.1% in the postevent cycle (**B**). COVID-19, coronavirus disease 2019.

**Fig. 3.**
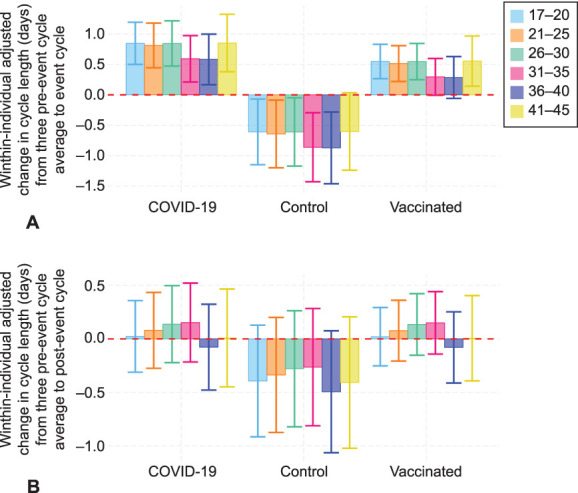
Adjusted within-individual change in cycle length from the average length of three cycles preevent and the event cycle (**A**) or the postevent cycle (**B**) across groups and age groups.

Individuals in the control group experienced a 0.68-day decrease in cycle length between cycles 1–3 and cycle 4 (95% CI −1.18 to −0.19, *P*=.007). Individuals in the vaccinated group experienced a 1.14-day unadjusted increase in cycle length during the first vaccination cycle compared with the prevaccination average (Table 2, 95% CI 0.63–1.66, *P*<.001). Individuals in the COVID-19 cohort experienced a 1.45-day unadjusted increase in cycle length during the first COVID cycle compared with the average of their three pre-COVID cycles (Table 2, 95% CI 0.89–2.02, *P*<.001). A Tukey HSD test revealed no significant differences between the vaccinated and COVID-19 groups (diff=0.31, 95% CI −0.05 to 0.67, *P*=.11), although they were both different from the control group. The overlaid histogram shows a cycle length change distribution in individuals in the COVID-19 and vaccinated groups that is roughly equivalent to that in the control group, although slightly right-skewed relative to the control distribution (Fig. 2A). The proportion of individuals who experienced a clinically significant change in cycle length of more than 8 days between the event cycle and the preevent average was 6.9% in the control group, 9.7% in the COVID-19 group, and 6.3% in the vaccinated group. After applying a Bonferroni correction, this proportion remained higher for the COVID-19 group (*P*<.001; Appendix 12, available online at http://links.lww.com/AOG/D312). As compared with the control group, after adjusting for age and country, the change in cycle length remains at 1.45 days for the COVID-19 cohort (95% CI 0.86–2.04, *P*<.001) and 1.14 days for the vaccinated cohort (95% CI 0.60–1.69, *P*<.001, Table 2, Fig. 3A).

**Table 2.**
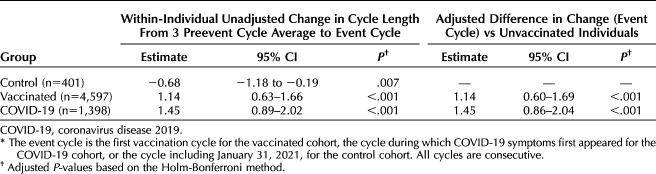
Unadjusted and Adjusted Estimates for Changes in Cycle Length Between the Three Preevent Cycle Average (Cycles 1–3) and the Event Cycle (Cycle 4) (n=6,396)*

*Alvergne. Menstrual Cycle Length and COVID-19. Obstet Gynecol 2023*.

*Alvergne. Menstrual Cycle Length and COVID-19. Obstet Gynecol 2023.*

Individuals in the COVID-19 cohort did not experience an increase in unadjusted cycle length cycle during the first post-COVID cycle compared with the three pre-COVID cycles (Table 3, 95% CI −0.12 to 0.96, *P*=.3), nor did individuals in the control group (95% CI −0.83 to 0.12, *P*=.3) or the vaccination group (95% CI −0.08 to 0.92, *P*=.3). The overlaid histogram shows a cycle length change distribution in individuals in the COVID-19 and vaccinated groups that is roughly equivalent to that in the control group (Fig. 2B). The proportion of individuals who experienced a clinically significant change in cycle length of more than 8 days was 4.7% in the control group, 8.1% in the COVID-19 group, and 6.9% in the vaccinated group. After applying a Bonferroni correction, these proportions were not statistically different across groups (*P*=.33; Appendix 13, available online at http://links.lww.com/AOG/D312). Adjusted changes postcycle were not different from unadjusted changes (Table 3, Fig. 3B).

**Table 3.**
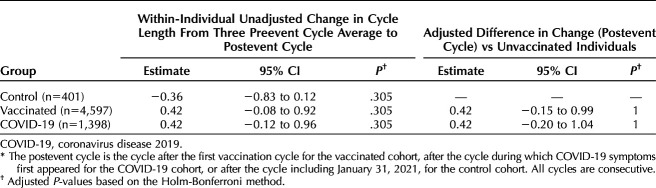
Unadjusted and Adjusted Estimates for Changes in Cycle Length Between the Three Preevent (COVID-19 or Vaccination) Cycle Average (Cycles 1–3) and the Postevent Cycle (Cycle 5) (n=6,396)*

We conducted a subanalysis in a cohort of individuals vaccinated at least 3 months before the onset of COVID-19 (n=2,335). Individuals in the cohort experienced a 1.02-day unadjusted increase in cycle length from the three prevaccination cycles compared with the first post-COVID cycle (95% CI 0.50–1.54, *P*<.001) (Appendix 5, http://links.lww.com/AOG/D312), which is lower than the change experienced by those in the unvaccinated cohort who had COVID-19 (1.44, 95% CI 0.90–1.99, *P*<.001) (Appendix 5, http://links.lww.com/AOG/D312).

A Tukey HSD test revealed a significant difference between the group of individuals who had COVID-19 after vaccination and the COVID-only group (diff=−0.42, 95% CI −0.85 to 0.0031, adjusted *P*=.05) but no differences between the vaccinated-only group and the COVID-19-after-vaccination group (diff=−0.17, 95% CI −0.15 to 0.49, adjusted *P*=.52). The overlaid histogram shows a cycle length change distribution in individuals in the COVID-19 and vaccinated groups that is roughly equivalent to that in the control group (Appendix 4, http://links.lww.com/AOG/D312). As compared with the control group, after adjusting for age and country, the change in cycle length is 1.02 days (95% CI 0.47–1.57, *P*<.001) (Appendix 5, http://links.lww.com/AOG/D312) for the cohort that was vaccinated before experiencing COVID-19. There is no significant increase in cycle length between the average of the three cycles before vaccination and the first cycle post-COVID (95% CI −0.52 to 0.50, *P*=.98) (Appendix 6, http://links.lww.com/AOG/D312).

After removing cases that occurred during the Omicron variant dominance (n=293), the estimates remained the same (Appendix 7, http://links.lww.com/AOG/D312). When imputed data on BMI were included in the adjusted model, estimates remained unchanged (Appendix 8, http://links.lww.com/AOG/D312). Using alternative timings for the artificial COVID cycle in the control group lead to significant and similar (0.82, 1.08 and 1.09-day increase) changes for the COVID-19 group but no changes for the control group and similar changes for the vaccinated group in some but not all cases (Appendix 9, http://links.lww.com/AOG/D312).

## DISCUSSION

We evaluated 32,570 cycles from 6,514 individuals to evaluate whether COVID-19 is associated with changes in menstrual cycle length and how any such changes might compare with COVID-19 vaccination or a control group. Among unvaccinated participants reporting having COVID-19, we found a 1.45-day increase in menstrual cycle length as compared with their three preevent cycle-length average. This change quickly resolved in the post-COVID cycle. We found a similar small increase in menstrual cycle length for the COVID-19–vaccinated cohort as compared with their three preevent cycle-length average, which also quickly resolved in the postvaccination cycle. Although the differences for the COVID-19 and vaccinated groups were each statistically different from the control group, they were not significantly different from one another. Changes in cycle length associated with COVID-19 and vaccination during the event cycle were small in magnitude and not clinically significant at the population level, but the proportion of individuals who experienced a clinically significant change of more than 8 days was higher for individuals with COVID-19 than for those in the vaccination or control groups. We also found that COVID-19 vaccination at least 3 months before the onset of COVID-19 symptoms was protective for COVID-19–associated changes in cycle length.

Existing literature on the effects of COVID-19 on the menstrual cycle is scant and somewhat variable, but the overall signal appears to be consistently small in magnitude. The Nurses' Health Study 3 showed no associations between COVID-19 and cycle length changes in a prospective study of 3,858 premenopausal U.S. health care professionals.^23^ This study's use of self-reported data collected 5–10 years apart^1^ likely hinders the resolution needed to detect small changes. Previous studies among individuals who tested positive for SARS-CoV-2 infection found larger effects, with 15–25% of individuals reporting changes to their menstrual cycles after SARS-CoV-2 infection.^16,28^ These studies likely overestimated the effects due to a lack of control group and a higher proportion of severely ill patients in the study populations.^1^ Yet, a biological effect is suggested by a small study of 73 mostly Black and Hispanic women showing a significant association between immunoglobulin G antibodies and the perception of menstrual irregularities.^22^ Our results are strengthened by the use of prospectively tracked menstrual cycles and a control group, the exclusion of contraceptive users, and the power to detect a 1-day difference in cycle length.

Several limitations exist in our study. First, we relied on app users' self-report of COVID-19 symptoms or a positive test result, as well as dates of infection or illness or vaccination. However, an individual's self-report of COVID-19 symptoms earlier in the pandemic, when we performed data collection, was shown to be highly correlated with COVID-19, and most individuals have easy access to dates of their COVID-19 vaccinations.^29,30^ Our control cohort likely included asymptomatic individuals with COVID-19, who may be similar to controls in that they likely do not experience menstrual cycle disturbances. A recent study supports this assumption, showing a positive association between the number of COVID-19 symptoms and menstrual cycle changes.^28^ Second, SARS-CoV-2 variants may act differently on menstrual cycles, and we have no biological data to assess this possibility, although removing the more virulent Omicron wave from our data did not change the results. Third, our menstrual data rely on tracking behavior, and any heterogeneity in tracking behavior makes it difficult to distinguish between a real biological effect and a change in tracking behavior due to being ill. We addressed this issue by including only *regular trackers* (ie, participants who tracked any symptom in the app in the 90 days after the start of the cycle during which symptoms or the vaccination occurred). Although this prevents us from including individuals who had simply forgotten to track their cycles, it excludes people who stopped tracking altogether because of being too symptomatic from COVID-19.

We also do not have additional information about this cohort's education, income, or ethnicity, but prior research using a cohort of period tracker app users from the United States shows that the demographics for respondents to surveys are reflective of the broader U.S. population.^31^ We cannot account for pandemic stress, but adjusting for pandemic stress has previously been found not to alter associations between cycle length and COVID-19 vaccination.^23^ We are unable to comment on how COVID-19 or vaccination affects hormonal contraceptive users or individuals with gynecologic diseases. Previous studies on vaccination and menstrual cycle length have shown that associations may be small to no increase in individuals with gynecologic disorders.^7–9,15^

In summary, although the proportion of individuals who experienced a clinically significant change in cycle length of more than 8 days was higher for those with COVID-19, at the population level, experiencing COVID-19 was associated with a small and temporary change in cycle length similar to that with COVID-19 vaccination.
